# Exogenous carnitine can modulate oxaliplatin sensitivity in colorectal cancer cells *in vitro*

**DOI:** 10.3389/fphar.2026.1963206

**Published:** 2026-10-28

**Authors:** Yufang Fan, Jian Huang, Liqiang Dai, Lifeng Huang, Yuemiao Chen, Hongkai Cai, Qiuru Zhou, Wei Jiang, Ziyi Zhang, Xiaoping Cai, Yao-Gen Shu

**Affiliations:** 1 Wenzhou Central Hospital, Wenzhou, Zhejiang, China; 2 Wenzhou Institute, University of Chinese Academy of Sciences, Wenzhou, Zhejiang, China

**Keywords:** carnitine, colorectal cancer, drug resistance, fatty acid oxidation, oxaliplatin

## Abstract

**Objective:**

The clinical management of colorectal cancer (CRC) is hampered by the frequent development of oxaliplatin (OXA) resistance. This study investigated whether exogenous L-carnitine (CAR) modulates OXA sensitivity in CRC cells and whether any such effect depends on fatty acid oxidation (FAO).

**Methods:**

The effects of carnitine, with or without OXA, on cell viability, proliferation, migration, oxygen consumption, apoptosis, cell-cycle distribution, and intracellular reactive oxygen species (ROS) were evaluated in HCT116 parental cells and an OXA-resistant subline (HCT116/L), using CCK-8, colony formation, wound healing, Seahorse XF analysis, flow cytometry, and ROS fluorescence probes.

**Results:**

*In vitro*, exogenous carnitine dose-dependently reduced CRC cell viability, proliferation, migration, and respiration, and promoted apoptosis. Co-treatment with carnitine partially attenuated the anti-proliferative and pro-apoptotic effects of OXA in HCT116/L cells, consistent with reduced chemosensitivity. This effect was accompanied by a partial restoration of fatty-acid-dependent respiration and a reduction in OXA-induced ROS accumulation.

**Conclusion:**

In OXA-resistant HCT116/L (MSI-H) cells, exogenous carnitine is associated with reduced OXA sensitivity, an effect that requires fatty acid oxidation. These findings identify carnitine-FAO metabolism as a potential determinant of OXA response; whether limiting carnitine availability can restore OXA sensitivity *in vivo* remains a hypothesis to be tested.

## 1 Introduction

Colorectal cancer (CRC) remains one of the leading causes of cancer-related morbidity and mortality worldwide (Park et al., 2017). According to the International Agency for Research on Cancer (IARC/WHO), CRC accounted for over 900,000 deaths globally in 2022 (Ferlay et al., 2024). Although survival rates have improved significantly for early-stage (I–II) disease, the 5-year overall survival rate for late-stage (III–IV) patients remains below 30%, compounded by a high risk of recurrence (Chatila et al., 2021). Platinum-based chemotherapy currently constitutes the cornerstone of CRC management (Singh et al., 2024). Oxaliplatin (OXA), a third-generation platinum compound, combined with fluoropyrimidine regimens is established as the standard first-line therapy for locally advanced and metastatic CRC (Adebayo et al., 2023). However, the clinical efficacy of OXA is frequently constrained by dose-limiting toxicities, including myelosuppression, gastrointestinal distress, and peripheral neuropathy (Alzahrani et al., 2021; Mármol et al., 2019). More critically, primary or acquired resistance severely limits therapeutic response, leading to poor prognosis in a substantial proportion of patients (Adlard et al., 2002; Wang and Guo, 2012). OXA, in combination with fluoropyrimidines (FOLFOX or CAPOX), is a standard first-line regimen for locally advanced and metastatic CRC and achieves objective response rates of approximately 45% ∼ 60% in first-line metastatic disease; in contrast, OXA is rarely re-introduced in the second-line setting, where irinotecan-based or targeted regimens are generally used (Tournigand et al., 2004). Consequently, elucidating the mechanisms underlying OXA resistance and developing effective chemosensitization strategies have become urgent priorities for improving patient outcomes.

Reactive oxygen species (ROS) serve as central regulators of cellular proliferation, apoptosis, and redox homeostasis (Zhao et al., 2023). Under physiological conditions, ROS function as signaling molecules; however, excessive accumulation disrupts redox balance, precipitating DNA damage, protein oxidation, and lipid peroxidation, which ultimately trigger programmed cell death (Bhattacharyya et al., 2014; D’Autréaux and Toledano, 2007; Bhattacharjee, 2019). OXA exerts its cytotoxic effects primarily by forming DNA adducts and inducing mitochondrial dysfunction, leading to pathological ROS overproduction (Alcindor and Beauger, 2011; Gorrini et al., 2013). To counteract chemotherapy-induced oxidative stress, tumor cells frequently upregulate endogenous antioxidant networks, such as the glutathione (GSH) pool and nicotinamide adenine dinucleotide phosphate (NADPH) regeneration systems (Jeon et al., 2012; Poprac et al., 2017; Li et al., 2018). Recent evidence identifies metabolic reprogramming as a pivotal driver of chemoresistance (Wang et al., 2024). Among these alterations, fatty acid oxidation (FAO) serves as a highly efficient energy-yielding pathway that not only generates substantial adenosine triphosphate (ATP) but also supplies critical reducing equivalents in the form of NADPH, thereby playing a decisive role in maintaining redox homeostasis (Qu et al., 2016; Schlaepfer et al., 2014; Carracedo et al., 2013; Zhou et al., 2014). Aberrantly upregulated FAO can directly promote tumor cell survival and drug tolerance through multi-dimensional mechanisms, including metabolic rewiring, signaling pathway modulation, and cell cycle alteration (Yu et al., 2024; Nassar et al., 2020; Li et al., 2022; Kuo and Ann, 2018; Ye et al., 2010; Le Borgne et al., 2017; Donisi et al., 2025; Ning et al., 2026).

The initiation of FAO is strictly dependent on the mitochondrial import of long-chain fatty acids (LCFAs) via the carnitine shuttle system. Initially, LCFA influx is mediated by membrane transporters such as CD36 (Schwenk et al., 2010; Rada et al., 2020). In the cytosol, free fatty acids are activated by acyl-CoA synthetases and converted to acyl-carnitines by carnitine palmitoyltransferase 1 (CPT1) on the outer mitochondrial membrane. Subsequently, the carnitine/acylcarnitine translocase (CACT) facilitates transport across the inner membrane, where CPT2 reconverts them into acyl-CoA for entry into the β -oxidation cycle (Ma et al., 2018; Bonnefont et al., 2004). Wang et al. (Wang et al., 2020) demonstrated that OXA treatment significantly upregulates CPT1B and CPT2 expression in CRC cells, enhancing FA catabolism and correlating with diminished chemosensitivity. This transport axis may thus serve as a predictive biomarker and a potential therapeutic target for reversing resistance. Their *in vitro* studies confirmed that specific knockdown of CPT2 or pharmacological inhibition of the FAO pathway effectively restores OXA sensitivity without inducing marked cytotoxicity. Multiple investigations consistently report that aberrant activation of the FAO pathway in gastrointestinal malignancies constitutes a major mechanism mediating OXA resistance (Lin et al., 2021; Wu et al., 2020; He et al., 2019).

As an essential cofactor for the carnitine shuttle, carnitine is indispensable for LCFA mitochondrial transport and subsequent β -oxidation. Derived from lysine and methionine biosynthesis (approximately 25% endogenously in the liver and kidneys) and dietary intake (approximately 75%), carnitine is ubiquitous across mammalian tissues (Sakai et al., 2017; Rabito et al., 2013). Notably, several anticancer agents have been shown to interfere with carnitine synthesis, intestinal absorption, or renal excretion. Carnitine is a necessary co-substrate of the carnitine shuttle and can become limiting for long-chain FAO, whereas CPT1-catalysed acylcarnitine formation is generally regarded as the committed, regulatory step of long-chain FAO (Bonnefont et al., 2004; Qu et al., 2016). Adequate carnitine levels not only sustain cellular energy demand but also exert cytoprotective effects by scavenging free radicals, preserving mitochondrial membrane integrity, and suppressing lipid peroxidation cascades (Farahzadi et al., 2016; Fathi et al., 2020; Fathi et al., 2021). Despite compelling evidence implicating FAO and LCFA transport in CRC chemoresistance, the precise mechanisms by which carnitine homeostasis regulates OXA-induced oxidative stress responses and therapeutic efficacy remain incompletely characterized.

Therefore, this study aimed to evaluate the impact of carnitine intervention on the biological behavior and drug sensitivity of OXA-resistant CRC cells, with particular emphasis on its role in maintaining mitochondrial redox homeostasis and mediating chemosensitization. The findings are expected to provide novel targeting strategies for overcoming OXA resistance in CRC and offer a rationale for clinical therapeutic optimization.

## 2 Materials and methods

### 2.1 Cell lines and culture

HCT116 (Meisen, ♯ CTCC-001-002) parental cells and their OXA-resistant subline [HCT116/L (Meisen, ♯ CTCC-0313-NY)] were purchased from MeisenCell (China). All cell lines were maintained in Dulbecco’s modified Eagle’s medium [DMEM(Meisen, ♯ CTCC-002-008)] supplemented with 10% fetal bovine serum [FBS (Meisen, ♯ CTCC-002-071)] at 37 ° C in a humidified atmosphere containing 5% ${\text{CO}}_{2}$ (Tran et al., 2017).

### 2.2 Reagents

L-carnitine (levocarnitine; ≥ 98% purity) (MCE, ♯ BS350B) was purchased from MedChemExpress (United States). DMEM and FBS were obtained from MeisenCell (China). The Cell Counting Kit-8 (CCK-8) assay kit (biosharp, ♯ BS350B), Propidium iodide (PI) staining kits (UELandy, ♯ P4055S), Annexin V-APC/7-AAD apoptosis kit (absin, ♯ abs50008), ROS detection kit (Solarbio, ♯ CA1410), and oxygen consumption rate (OCR) assay kit (Agilent, ♯ 103015-100) were sourced from Biosharp (China), Uelandy (China), Absin (China), Solarbio (China), and Agilent (China), respectively.

### 2.3 Cell viability assay (CCK-8)

Cells ( $5\times 10^{3}$ per well) were seeded in 96-well plates and treated according to experimental design. Following reagent addition, plates were incubated for 1 h. Absorbance was measured at 450 nm using an enzyme-linked immunosorbent assay (ELISA) reader (Assay and Assay, 2013).

### 2.4 Colony formation assay

Cells ( $5\times 10^{2}$ per well) were seeded in 6-well plates. After about 14 days of treatment, colonies were fixed with 4% paraformaldehyde for 15 min, stained with 0.1% crystal violet for 30 min, washed with PBS, and counted under a light microscope (Franken et al., 2006).

### 2.5 Wound healing assay

Confluent monolayers ( ≥ 90%) were scratched using a sterile pipette tip, followed by serum-free medium incubation for 0–48 h. Migration progress was recorded via microscopy (Chen, 2012).

### 2.6 Oxygen consumption rate measurement

Fatty acid oxidation (FAO) assay. Substrate-specific FAO was assessed with the Seahorse XF Palmitate Oxidation Stress Test Kit (Agilent, ♯ 103015-100). The day before the assay, growth medium was replaced with substrate-limited medium (DMEM supplemented with 0.5 mM glucose, 1 mM L-glutamine, 1% FBS (Meisen, ♯ CTCC-002-071) and 0.5 mM L-carnitine (MCE, ♯ BS350B)), and the cells were incubated overnight at 37 ° C, 5% ${\text{CO}}_{2}$ . On the day of the assay, cells were washed once and replenished with FAO assay medium [Seahorse XF DMEM, pH 7.4, supplemented with 2.5 mM glucose, 0.5 mM L-carnitine and 5 mM HEPES; pH adjusted to 7.4], 180 μ L per well, and equilibrated at 37 ° C in a ${\text{CO}}_{2}$ -free incubator for 45 ∼ 60 min. Immediately before the run, palmitate-BSA (final palmitate concentration 500 μ M), BSA vehicle, or etomoxir (final concentration 4 μ M; MedChemExpress, ♯ HY-50202) was added to the designated wells and the cells were incubated for a further 20 min. FAO was then measured following the sequential injection of oligomycin (1.5 μ M), FCCP (1.0 μ M) and rotenone/antimycin A (0.5 μ M each) (Zhao et al., 2022).

### 2.7 Cell cycle analysis

After propidium iodide staining, ≥ 20,000 events per sample were acquired on a Beckman Coulter flow cytometer and analysed with FlowJo V10.9.0 flow-analysis software. The hierarchical gating was: (i) FSC-A/SSC-A singlets gate to exclude cellular debris and free nuclei; (ii) FSC-A/FSC-H singlet gate to remove cell doublets; (iii) a second PI-A/PI-W singlet gate, applied specifically in the DNA channel, to exclude residual doublets whose DNA content could otherwise be misclassified as G2/M; and (iv) a single-parameter PI histogram in which G0/G1, S and G2/M phases were deconvolved using the Dean-Jett-Fox model, with the G2 peak constrained to twice the G1 mean and G0/G1 coefficient of variation < 5%. Sub-G1 events (debris/apoptotic fragments) were excluded from the cell-cycle distribution and are not included in the reported percentages (Babes et al., 2018).

### 2.8 Apoptosis detection

After Annexin V-APC/7-AAD staining, cells were acquired and gated sequentially through: (i) FSC-A/SSC-A to exclude debris; (ii) FSC-A/FSC-H to exclude doublets; and (iii) the Annexin V/7-AAD dot plot. The reported apoptotic fraction is the sum of early (Annexin V+/7-AAD-) and late apoptosis (Annexin V+/7-AAD+); necrotic events (Annexin V-/7-AAD+) were not counted as apoptotic but are reported in the supplementary materials. Dead cells were therefore not “excluded” from the apoptosis readout—they are the informative population—but debris and doublets were removed before quantification (Alshehade et al., 2024).

### 2.9 Intracellular ROS measurement

Cellular ROS levels were detected using the 2′,7′-Dichlorodihydrofluorescein diacetate (DCFH-DA) probe according to the manufacturer’s protocol. Fluorescence intensity was quantified by flow cytometry (Beckman Coulter, United States) (Kim and Xue, 2020).

### 2.10 Statistical analysis

All experiments were performed in triplicate ( n=3 independent biological replicates). Data are presented as mean ± standard deviation (SD). Normality of distribution was assessed using the Shapiro-Wilk test, and homogeneity of variance was evaluated using Brown-Forsythe test. Multi-group comparisons were analyzed by one-way ANOVA followed by Tukey’s *post hoc* test (Graph-Pad Prism software, version 9.0; GraphPad Software, Inc.). Exact pairwise comparisons are specified in each figure. The four experimental groups were designated as Control (control group), Car (carnitine alone), Oxa (oxaliplatin alone), and Oxa + Car (co-treatment of oxaliplatin and carnitine), respectively. Statistical significance was defined as ∗p<0.05 , ∗∗p<0.01 , and ∗∗∗p<0.001 , ∗∗∗∗p<0.0001 ; ns indicates no significant difference.

## 3 Results

### 3.1 Exogenous carnitine and OXA suppress CRC cell viability and proliferation

To evaluate OXA sensitivity, HCT116 and HCT116/L cells were treated with OXA ( 6μ M) and assessed via CCK-8 and colony formation assays. CCK-8 results (Figure 1A) revealed a time-dependent suppression of cell viability by OXA. At 72 h, viable HCT116 cells decreased to 19.33%, whereas HCT116/L maintained significantly higher viability at 67.91%. Colony formation assays (1–2 weeks) further corroborated these findings (Figure 1B): OXA markedly inhibited clonogenic growth in parental cells (80 colonies vs. 370 control); notably, the HCT116/L subline exhibited significantly enhanced tolerance, retaining a substantially higher colony count (227 vs. 340 control) despite identical drug exposure. These data confirm that the commercially obtained HCT116/L subline retained its OXA-resistant phenotype under our culture conditions.

**FIGURE 1:**
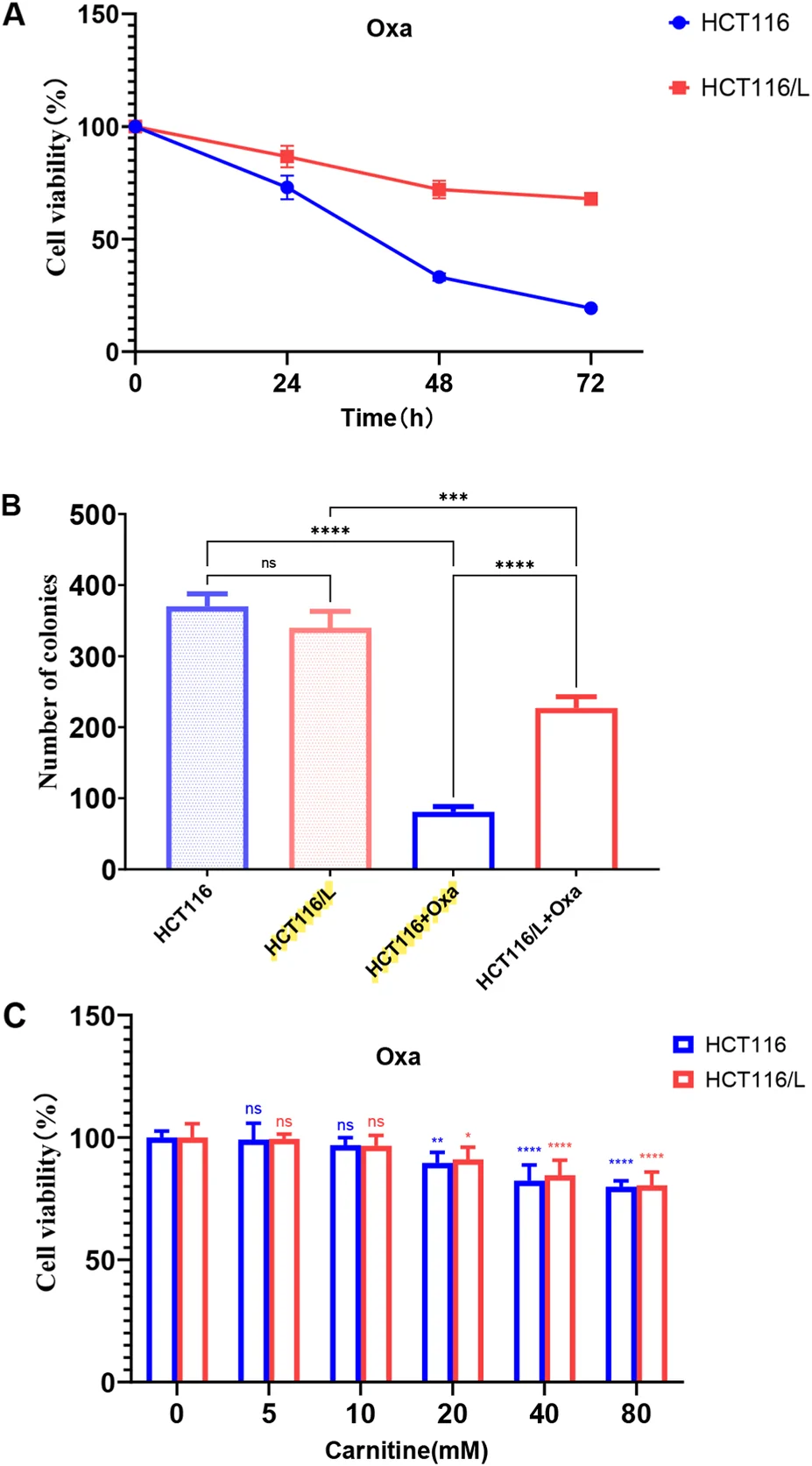
Oxaliplatin and exogenous carnitine inhibit CRC cell viability and proliferation. **(A)** CCK-8 assay showing time-dependent viability reduction in HCT116 and HCT116/L cells following OXA treatment. **(B)** Colony formation assay demonstrating colony number suppression by OXA. **(C)** Dose-dependent reduction in cell viability following 48 h carnitine exposure (Statistical significance denotes differences compared to the 0-dose control group). Oxaliplatin concentration is 6μ M. Data represent mean ± SD of three independent experiments. Statistical significance was determined by one-way ANOVA with Tukey’s *post hoc* test. ∗p<0.05 , ∗∗p<0.01 , ∗∗∗p<0.001 , ∗∗∗∗p<0.0001 ; ns, not significant.

To assess the impact of carnitine alone, cells were treated with increasing concentrations (5 ∼ 80 mM) for 48 h. CCK-8 assays demonstrated that exogenous carnitine dose-dependently reduced viability in both lines: cell viability declined from 99.16% at 5 mM carnitine concentration to 79.83% at 80 mM carnitine concentration in HCT116 cells, and from 99.42% at 5 mM carnitine concentration to 80.41% at 80 mM carnitine concentration in HCT116/L cells (Figure 1C).

### 3.2 Carnitine attenuates OXA sensitivity in resistant cells

HCT116/L cells were co-treated with carnitine (20 mM) and OXA ( 6μ M). CCK-8 assays showed that viability dropped to 92.06% with carnitine alone, 73.13% with OXA alone, and 84.1% in the combination group (Figure 2A). Colony formation similarly revealed a protective effect of carnitine: colonies decreased from 253 (control) to 241 (carnitine), 170 (OXA), and rebounded to 214 (combination; Figure 2B). Wound healing analysis demonstrated that OXA significantly inhibited cell migration under low-proliferation conditions (migration rate at 48 h: 31.31% vs. 50.78% in the control group). Compared with OXA monotherapy, the combination treatment group exhibited a marked increase in migration rate (38.17%), indicating a partial restoration of migratory capacity and suggesting that carnitine attenuated the anti-migratory effect of OXA (Figure 3A). Seahorse XF analysis indicated that OCR decreased from 108.7 pmol/min (control) to 100.3 pmol/min (carnitine), 78.9 pmol/min (OXA), and increased to 93.8 pmol/min in the combination group (Figure 3B). Cell cycle profiling revealed that carnitine alone did not significantly alter distribution: G1 phase shifted from 74.83% to 73.86%, S phase from 14.05% to 14.75%, and G2/M from 11.13% to 11.45%. OXA monotherapy induced robust G2/M arrest (33.99%), with corresponding G1 and S phases at 61.35% and 4.66%. Combination treatment weakened this arrest, yielding 65.35% G1, 3.48% S, and 31.16% G2/M (Figure 4A). Apoptosis assays paralleled these trends: rates increased from 2.27% (control) to 8.06% (carnitine), 27.1% (OXA), and 18.27% (combination; Figure 4B). Collectively, these results indicate that exogenous carnitine counteracts OXA-induced cytotoxicity, proliferation arrest, and apoptosis in HCT116/L cells.

**FIGURE 2:**
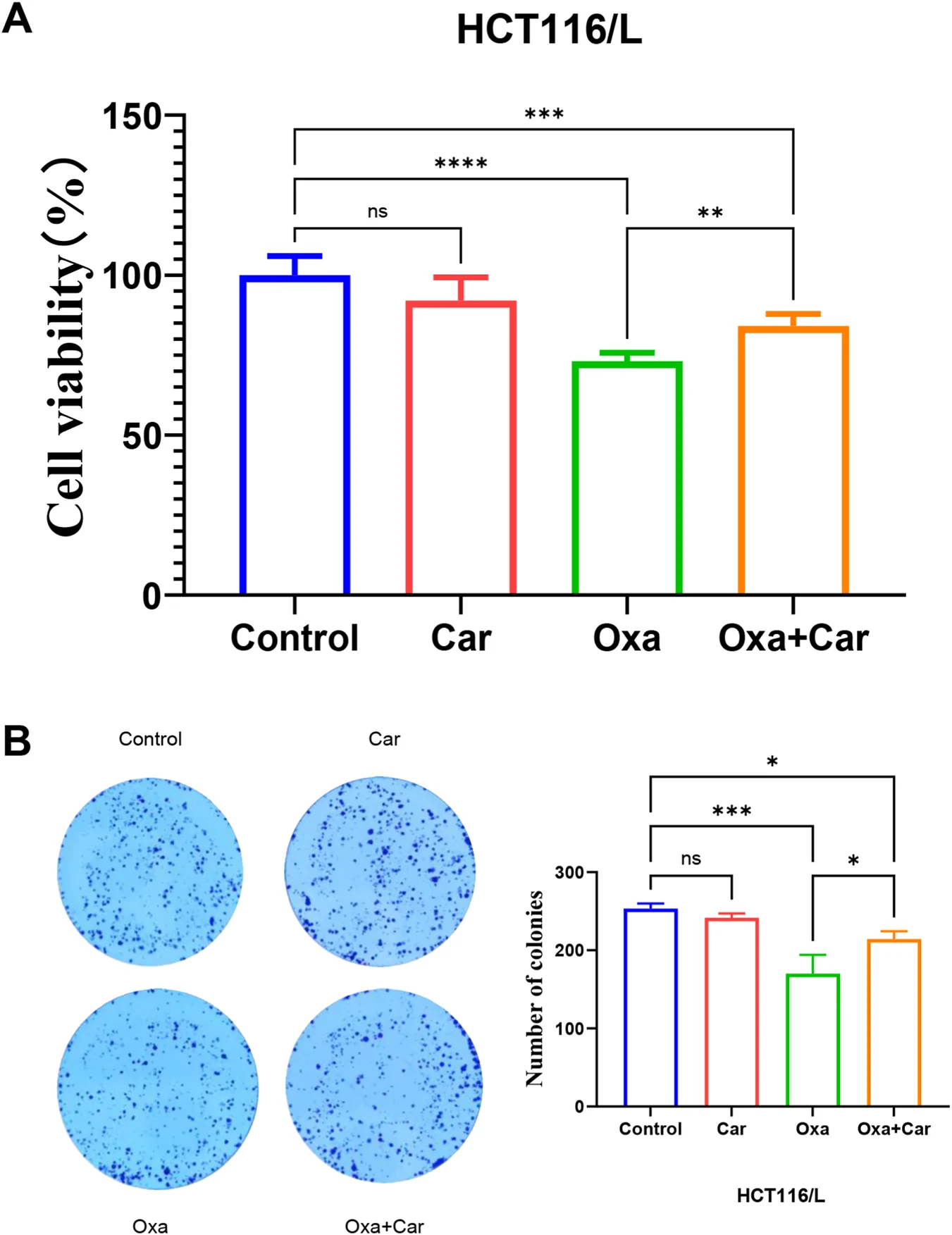
Carnitine attenuates OXA-induced cytotoxicity in resistant cells. **(A)** CCK-8 viability for HCT116/L cells treated with control, carnitine, OXA, or combination. **(B)** Colony formation counts reflecting synergistic/protective effects. Oxaliplatin and carnitine concentrations are 6μ M and 20 mM, respectively. Data represent mean ± SD of three independent experiments. Statistical significance was determined by one-way ANOVA with Tukey’s *post hoc* test. ∗p<0.05 , ∗∗p<0.01 , ∗∗∗p<0.001 , ∗∗∗∗p<0.0001 ; ns, not significant.

**FIGURE 3:**
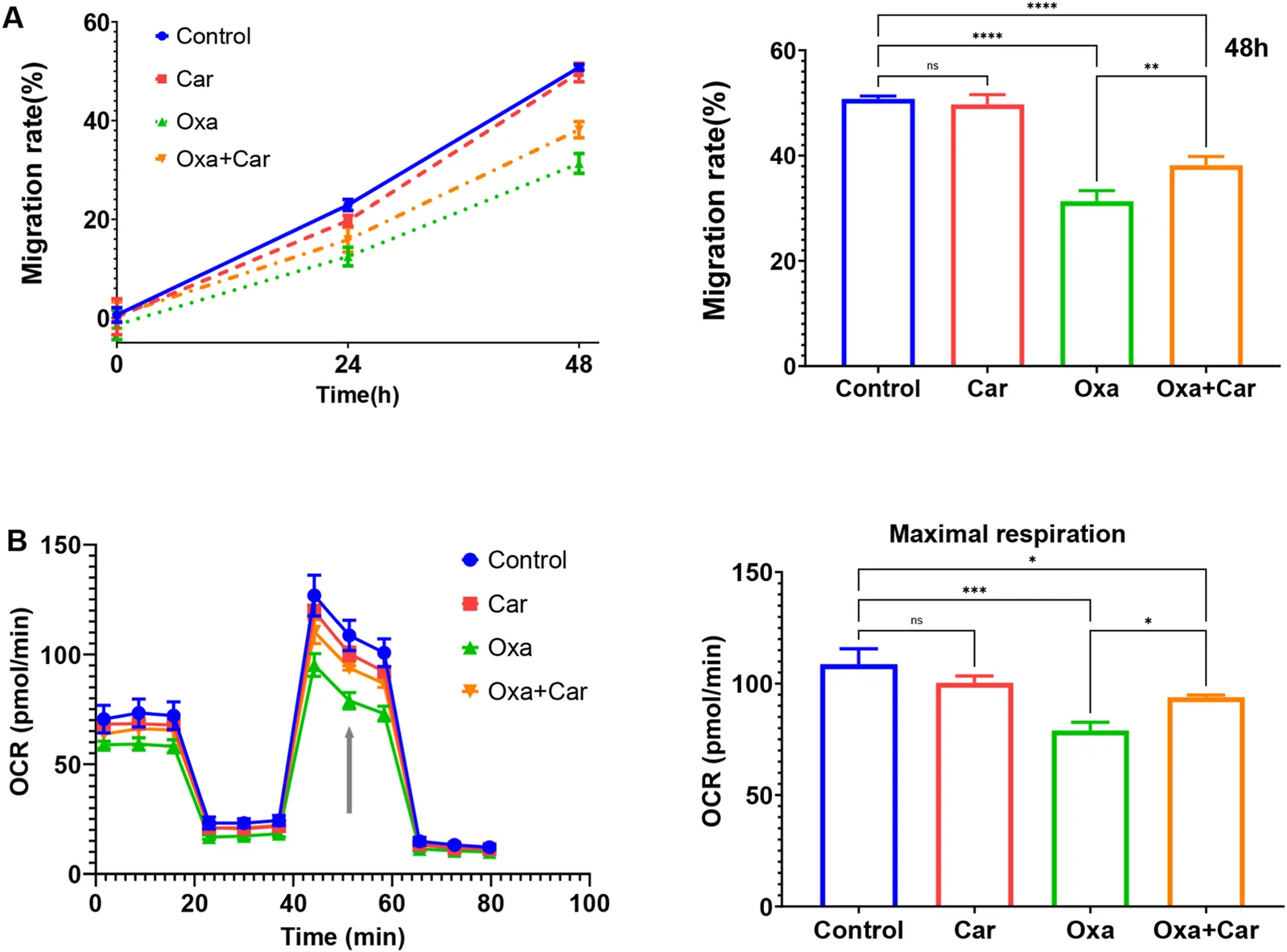
Carnitine modulates migration and mitochondrial respiration in HCT116/L cells. **(A)** Wound healing assay quantifying migratory capacity recovery upon combination treatment. **(B)** Seahorse XF-derived OCR measurements indicating FAO flux alterations. Data normalized to protein content. Oxaliplatin and carnitine concentrations are 6μ M and 20 mM, respectively. Data represent mean ± SD of three independent experiments. Statistical significance was determined by one-way ANOVA with Tukey’s *post hoc* test. ∗p<0.05 , ∗∗p<0.01 , ∗∗∗p<0.001 , ∗∗∗∗p<0.0001 ; ns, not significant.

**FIGURE 4:**
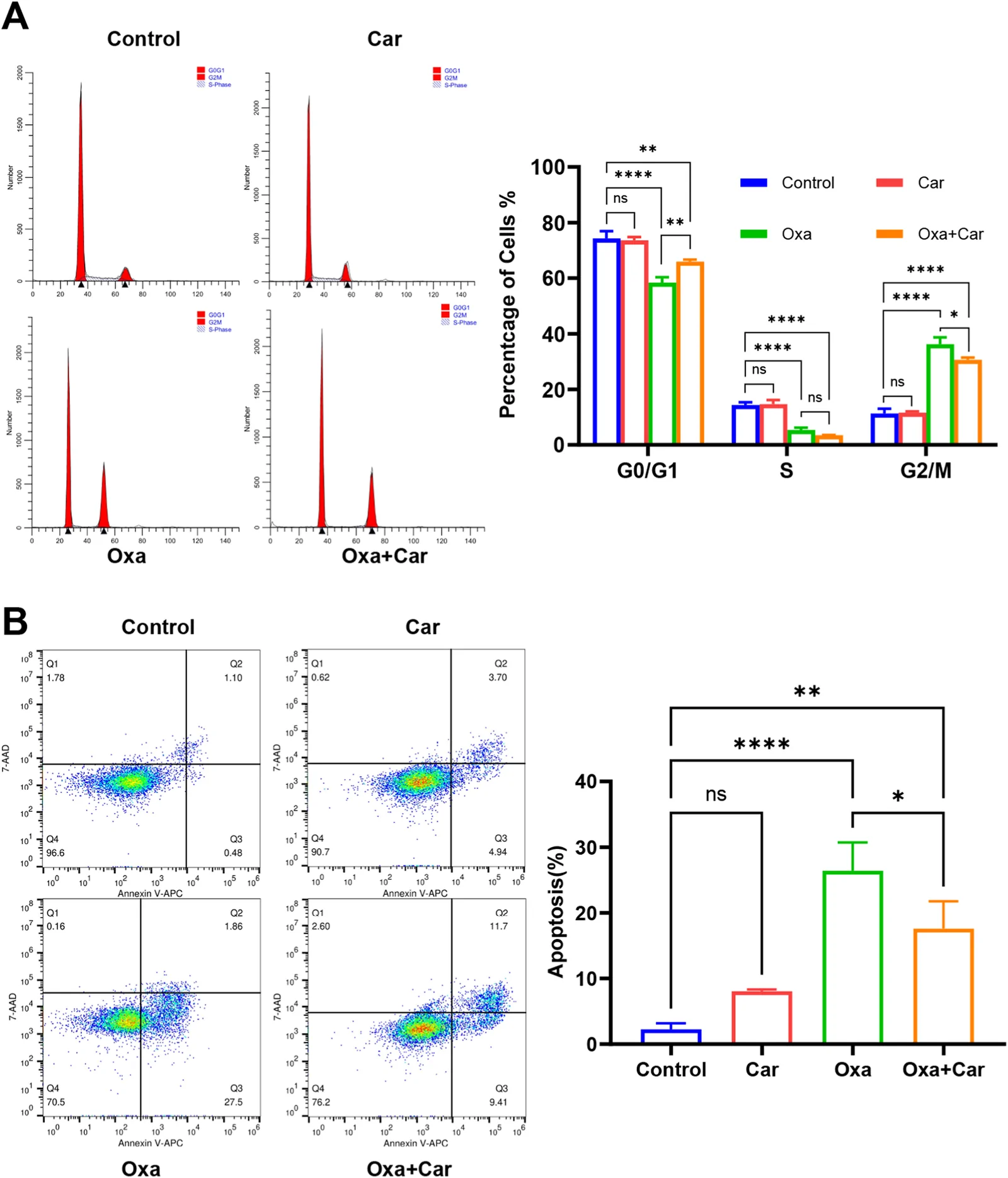
Carnitine alters cell cycle distribution and apoptosis rates under OXA stress. **(A)** Flow cytometry profiles showing G2/M phase attenuation in combination group. **(B)** Annexin V-APC/7-AAD staining quantifying early/late apoptotic populations. Oxaliplatin and carnitine concentrations are 6μ M and 20 mM, respectively. Data represent mean ± SD of three independent experiments. Statistical significance was determined by one-way ANOVA with Tukey’s *post hoc* test. ∗p<0.05 , ∗∗p<0.01 , ∗∗∗p<0.001 , ∗∗∗∗p<0.0001 ; ns, not significant.

### 3.3 Carnitine modulates chemoresistance via ROS regulation

Given that ROS mediate OXA-induced apoptosis and FAO generates NADPH to buffer oxidative stress, we investigated whether carnitine influences intracellular ROS dynamics. Following nutrient deprivation and exogenous fatty acid stimulation, DCFH-DA probe staining combined with flow cytometry revealed that carnitine supplementation slightly reduced baseline ROS levels in HCT116/L cells. OXA treatment caused marked ROS accumulation, which was partially attenuated upon co-treatment with carnitine (Figure 5). These findings suggest that carnitine-facilitated FAO enhances cellular antioxidant capacity, thereby mitigating OXA-triggered oxidative stress and promoting drug tolerance.

**FIGURE 5:**
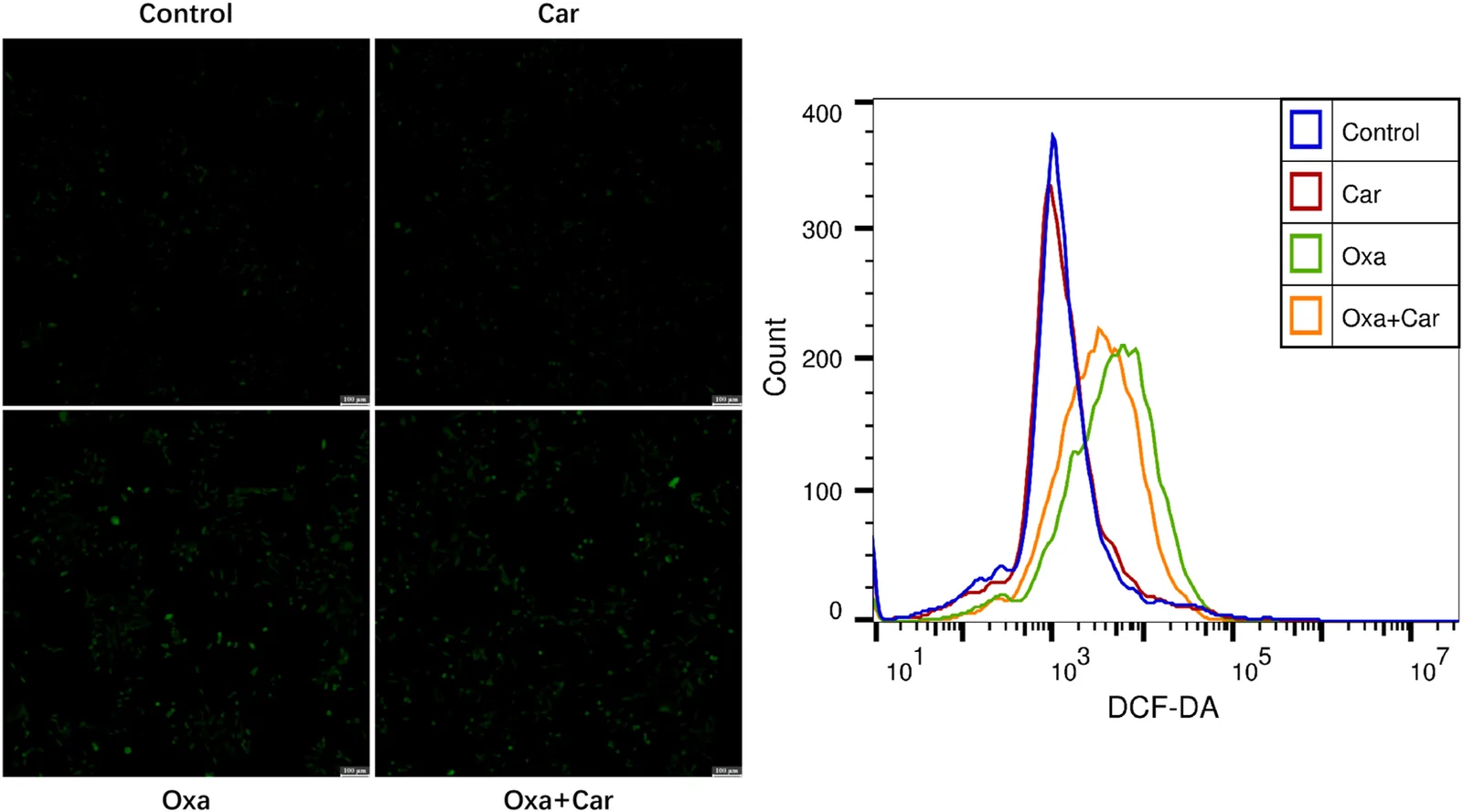
Carnitine mitigates OXA-induced ROS accumulation via FAO enhancement. DCFH-DA fluorescence intensity measured by flow cytometry following fatty acid stimulation and drug treatment. Reduced ROS correlates with enhanced metabolic resilience. Oxaliplatin and carnitine concentrations are 6μ M and 20 mM, respectively.

## 4 Discussion

OXA remains a foundational chemotherapeutic agent for CRC; however, acquired resistance is the primary driver of treatment failure in metastatic and recurrent disease. Enhanced FAO has been consistently linked to improved OXA efficacy suppression, as upregulated fatty acid catabolism elevates intracellular NADPH pools, bolstering antioxidant defenses against OXA-induced ROS generation. Pharmacological or genetic disruption of FAO synergizes with OXA to amplify oxidative damage and suppress tumor proliferation. Carnitine, as a critical shuttle component, enables LCFA mitochondrial import for β -oxidation and ATP production. Our *in vitro* data demonstrate that while carnitine supplementation independently exerts growth-inhibitory and pro-apoptotic effects on CRC cells, its co-administration with OXA paradoxically attenuates chemosensitivity in resistant HCT116/L cells. This implies that modulating carnitine availability may serve as a viable strategy to restore drug responsiveness. HCT116 is a mismatch-repair-deficient, microsatellite-unstable (MSI-H) cell line, and generalizing our conclusions across both MSI and MSS phenotypes requires further validation.

Approximately 75% of systemic carnitine is derived from dietary sources, with the remaining 25% synthesized endogenously in hepatic and renal tissues via lysine and methionine metabolism. Dietary restriction represents a non-invasive approach to limit carnitine availability. Additionally, N,N,N-trimethyl-5-aminopentanoic acid (TMAVA), a trimethylated betaine compound produced by gut microbiota from trimethyllysine (TML) and found in certain foods, has been shown to substantially lower circulating carnitine levels (Zhao et al., 2022). Mechanistically, TMAVA competitively inhibits γ -butyrobetaine dioxygenase (BBOX), blocking endogenous synthesis, while concurrently suppressing OCTN2-mediated carnitine uptake in a concentration-dependent manner. Epidemiological data indicate that fecal TMAVA concentrations are significantly elevated in healthy individuals compared to CRC patients, and higher stool TMAVA correlates with an approximate threefold reduction in colorectal cancer risk (Goedert et al., 2014). Nevertheless, clinical application of TMAVA requires careful consideration due to its documented association with cardiac hypertrophy and impaired ventricular function (Zhao et al., 2022).

## 5 Limitations

This study was confined to *in vitro* models. While exogenous carnitine manipulation successfully delineated its impact on OXA sensitivity, future investigations should employ nude mouse xenograft or patient-derived organoid models to evaluate whether dietary modulation or pharmacological suppression of carnitine transport/biosynthesis translates into reduced tumor burden and enhanced chemosensitivity *in vivo*. Furthermore, validation across diverse CRC genetic backgrounds beyond the HCT116 lineage is necessary to establish broader therapeutic relevance.

## 6 Conclusion

Exogenous carnitine partially reversed the oxaliplatin-induced reductions in cell viability, G2/M arrest, apoptosis, and intracellular ROS accumulation in oxaliplatin-resistant colorectal cancer cells. This indicates that carnitine-associated metabolic reprogramming contributes to acquired oxaliplatin resistance, rather than being merely a secondary metabolic consequence.

These phenotypic findings are consistent with several recent lines of evidence and support three converging arguments. First, they identify a metabolism-centred node that operates in parallel to upstream signalling pathways such as the ${COX-2/PGE}_{2}$ /EP4 axis, whose pharmacological inhibition restores oxaliplatin sensitivity by aggravating oxidative stress (Huang et al., 2019); resistance may therefore be modulated not only by kinase or receptor inhibition, but also by nutrient availability and mitochondrial substrate utilization. Second, they are directionally concordant with genetic evidence that silencing or pharmacological inhibition of CPT1A reverses oxaliplatin resistance by suppressing FAO (Lin et al., 2021), lending biological plausibility to a carnitine-FAO-resistance axis. Third, they can be accommodated within the broader frameworks of proteostatic remodelling (Quan et al., 2023) and lipid-metabolic and ferroptotic regulation (Zeng et al., 2023; Di Blasio et al., 2026) in chemoresistant colorectal cancer: by modulating mitochondrial fatty acid flux, carnitine may alter the composition of peroxidation-susceptible lipid species and the NADPH and GSH buffering capacity, thereby shifting the threshold for oxaliplatin-induced ferroptosis.

These mechanisms nevertheless remain a working hypothesis. FAO flux, ${NADPH/NADP}^{+}$ and GSH/GSSG ratios were not directly measured, and CPT1 involvement was not functionally validated. Translational proposals such as dietary carnitine restriction therefore await *in vivo* pharmacological testing, validation across microsatellite stable (MSS) and microsatellite instability (MSI) subtypes, and safety assessment in normal tissues.

Collectively, this work adds a previously underappreciated dimension—carnitine availability and carnitine-dependent mitochondrial metabolism—to the understanding of metabolic adaptation and therapeutic failure in oxaliplatin-resistant colorectal cancer.

## Data Availability

The original contributions presented in the study are included in the article/supplementary material, further inquiries can be directed to the corresponding authors.
